# Preparation and Characterization of UV-Curable Acrylic Membranes Embedding Natural Antioxidants

**DOI:** 10.3390/polym12020358

**Published:** 2020-02-06

**Authors:** Ewa Rajczak, Bartosz Tylkowski, Magda Constantí, Monika Haponska, Boryana Trusheva, Giulio Malucelli, Marta Giamberini

**Affiliations:** 1 Faculty of Chemistry, Adam Mickiewicz University in Poznan, Uniwersytetu Poznanskiego 8, 61-614 Poznan, Poland; ewa.rajczak@amu.edu.pl; 2 Politecnico di Torino—Dipartimento di Scienza Applicata e Tecnologia, Viale Teresa Michel 5, 15121 Alessandria, Italy; giulio.malucelli@polito.it; 3 Eurecat, Centre Tecnològic de Catalunya, C/Marcel·lí Domingo, 43007 Tarragona, Spain; bartosz.tylkowski@eurecat.org (B.T.); monika.haponska@eurecat.org (M.H.); 4 Department of Chemical Engineering (DEQ), Universitat Rovira i Virgili, Av. Països Catalans, 26, 43007 Tarragona, Spain; magdalena.constanti@urv.cat; 5 Institute of Organic Chemistry with Centre of Phytochemistry, Bulgarian Academy of Sciences, 1113 Sofia, Bulgaria; bobi_tru@orgchm.bas.bg

**Keywords:** acrylic membrane, tea powder, propolis, antibacterial activity

## Abstract

We examine the behaviour of acrylic resin-based membranes containing natural anti-oxidants, such as *Galla chinensis* tea powder extract (TP) and Taiwanese green propolis (TGP), in different concentrations ranging between 5 and 20 wt %. Membrane morphology was investigated by means of Environmental Scanning Electron Microscopy (ESEM), while the UV-curing reaction was monitored by Fourier-Transform Infra-red (FTIR) spectroscopy. In most cases Thermogravimetric (TG), Differential Scanning Calorimetric (DSC) and Dynamo-mechanical Thermal (DMT) analyses showed that the desirable characteristics of the UV-cured acrylic resin are not substantially altered by the presence of the organic fillers. The release kinetics of polyphenols and flavonoids, determined in water for TP-containing membranes (ETx) and in ethanol/water mixture (7:3 *v*/*v*) for TGP-containing ones (EPx), was satisfactory, reaching a *plateau* after 24 h. Finally, preliminary antibacterial tests against *S. epidermidis* were performed on the membranes with higher additive amount and gave positive results for ET-type; on the contrary, no inhibitory effect was observed for the tested EP-type membranes.

## 1. Introduction

In recent years scientists have been putting growing attention to natural products, such as tea leaves and honeybee product called propolis; actually, these products have shown considerable antibacterial, antifungal, antiviral, anti-inflammatory and antioxidant activity, due to their high content of polyphenols and flavonoids as well as their derivatives [1,2]. Propolis is a natural resin collected by honeybees from plant parts and subsequently elaborated by them in order to seal and protect beehives [3]. Propolis composition comprises around 45% resins, 30% waxes and fatty acids, 10% essential oils, 5% pollens and 10% organic compounds and minerals [4]. It is well known for its anti-microbial properties, thus it can reduce biofilm generation resulting in accelerated healing processes [5]. Moreover, it may be used to develop anti-inflammatory drugs, i.e., for leukocyte recruitment inhibitory [6]. In particular, Taiwanese green propolis (TGP) reveals a broad spectrum of biological activities: propolins from Taiwanese green propolis inhibited the growth of Gram-positive bacterial strains like *Staphylococcus aureus*, *Listeria monocytogenes*, *Bacillus subtilis* and *Paenibacillus larvae* [7]. Moreover, propolis was proven to alleviate the toxic effect of bisphenol A and enhance the growth performance and biochemical variables of Nile tilapia [8].

Tea *Galla chinensis* reveals not only anti-oxidant properties but also anti-carious effects [9]. The tea powder was mainly used orally as a solution in the inhibition of enamel demineralization or remineralization of initial dental caries in vivo [10,11]. Recent research put an attention on tea polyphenol-mediated cancer prevention [12] and on the therapeutic properties of green tea against environmental toxins and toxicants (i.e., pesticides, mycotroxins or arsenic) [13].

Both tea and propolis are widely used in food industry as natural preservatives [14]. Moreover, given the strong biological potential of these products, extensive research is in progress, concerning their incorporation into membranes and polymeric materials, to both protect these natural products from early oxidation and inactivation and allow their gradual release, as well as for them to perform antioxidant and antibacterial activity towards the polymer itself. For instance, they were applied as antimicrobials and antioxidants in natural rubber [15,16]. An incorporation of natural anti-oxidant products into i.e., thin films gives a possibility to various application in medicine and industry. Until now, scientists proclaimed a wide range of tea and propolis-based formulations that exhibit benefit properties.

For instance, preparation and properties of polylactic acid (PLA) are greatly enhanced after adding TP (tea polyphenol) and CS (chitosan); an increase in the heat-sealing strength, water vapour permeability and solubility is observed [17]. When used for the preservation of cherries, the membrane with the mass ratio of 3:7 (TP:CS) can visibly extend the expiration date from two to eight days at room temperature. Edible films of pectin (PEC) and konjac glucomannan (KGM) were enriched with tea polyphenol (TP) in addition of 1–5 wt % [18]. The loading up to 2% created a stable and compact microstructure that increased the thermal stability of PEC/KGM/TP film. Additionally, TP endowed the film with better antioxidant and antibacterial activity as caused an inhibition of a common pathogen—Gram-negative bacterium *Escherichia coli*.

The addition of propolis extract to the silicone matrix coating yields anticorrosion protection of carbon steel. What is interesting, antibacterial tests revealed that films formulated with propolis were able to decrease the adhesion of the Gram-positive bacteria *S. aureus* [19]. Another recent, very interesting approach, was presented by Al-Bayaty et al. [20]. Biodegradable chips made from malaysian propolis embedded in chitosan base were checked according to their physical, biological and antibacterial properties. What is more, the formulations were tested for biodegradability towards trypsin. Scientist accomplished satisfactory level; the minimum inhibitory concentration (MIC) value of the propolis extraction against gram positive bacteria was 0.152 mg/mL. Natural rubber latex membranes were enhanced with three different types of propolis (red, green and poplar) [21]. Cytotoxic assays showed that in the level of 30% and 50% eluates, the membranes with red and poplar propolis were not toxic to fibroblast cells.

In the literature one can find other propolis formulation based on other matrices, e.g., alginate, agar [22], poly(vinyl alcohol) (PVA) [23,24] or chitosan varnishes [25].

Another approach is focused on networks created with anti-oxidants and thermosetting plastics. To the best to our knowledge, only few papers concerning this topic exist. Benyahya et al. performed a functionalization of the natural green tea tannins extract and catechins with epichlorohydrin and obtained the epoxy pre-polymers [26]. Heat-cured bio-based epoxy polymers were characterized by their high crosslinking density but their thermal stability was lower than the reference material (diglycidyl ether of bisphenol A). In another approach, Basnet et al. created tea catechin-based epoxy networks too, but formulated them with methanol soluble lignin extracted form eucalyptus [27]. This resulted in improved thermal stability of the thermosets.

Herein, we present new formulations based on anti-oxidants (tea extract form *Galla chinensis* or Taiwanese green propolis) and bisphenol-A etoxylate diacrylate UV-curable resin. The casted membranes were filled with 5, 10, 15 and/or 20 wt % of the powders. In case of new networks, it is required to check the influence of the filler on the mechanical and thermal properties of the polymer matrix; therefore, SEM, TG, DSC and DMT analyses were incorporated into the study. Then, the obtained membranes were evaluated by detailed release tests of polyphenols and flavonoids in time-dependent experiments. Finally, preliminary antibacterial activity tests against *Staphylococcus epidermidis* were performed on the membranes containing the highest additive amount in their formulations. 

## 2. Materials and Methods 

Folin–Ciocalteu’s reagent, sodium carbonate, aluminium chloride, peptone from soybean meal, enzymatic digest, beef extract powder for microbiology, gallic acid, and quercetin were purchased from Sigm-Aldrich (Sigma Aldrich Química, Madrid, Spain). Agar and sodium chloride were purchased from Scharlab (Scharlab S.L., Barcelona, Spain) and Fischer Scientific (Fisher Scientific Spain, Madrid, Spain), respectively. All reagents were used without further purification. *Staphylococcus epidermidis* (*S. epidermidis*, strain CECT231) was purchased from Colección Española de Cultivos Tipo (CECT, Valencia, Spain). Ethanol was supplied by Merck (Merck Life Science S.L.U, Madrid, Spain). Water was distilled using MilliQ Merck purification system (Merck Life Science S.L.U, Madrid, Spain).

**Figure 1:**
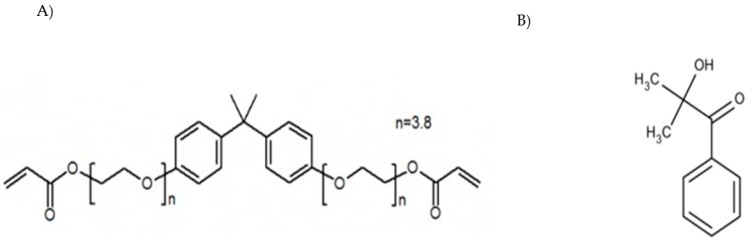
Chemical structures of Ebecryl 150 (**A**) and Irgacure 1173 (**B**).

The UV-curable acrylic resin Ebecryl 150 (bisphenol-A etoxylate diacrylate, referred as Eb), was purchased from Cytec Industries BV (Vlaardingen, The Netherlands). A photoinitiator (Irgacure 1173, 2,2-dimethyl-2-hydroxy acetophenone, Darocure MBF) was received from BASF company (Basf Italia Srl, Turin, Italy). The structures of these compounds are reported in Figure 1. 

Tea extract was obtained from commercially available *Galla chinensis* by mixing for 24 h in distilled water in the ratio of 5:1 (*v*/*w*). The solution was filtered and the obtained crude product was dried for 72 h at room temperature. Taiwanese green propolis (TGP) was harvested by a propolis collector in 2014 in Taiwan and then stored at 5 °C in darkness at the Institute of Organic Chemistry with Center of Phytochemistry at the Bulgarian Academy of Science in Sofia, Bulgaria. Before the membrane preparation the propolis sample was crushed using a rotating blade coffee grinder Bosch MKM6003 type KM13 (BSH Electrodomésticos España S.A, Madrid, Spain) to the dimension ≤ 100 µm and used as obtained.

### 2.1. Membrane Preparation

To produce the polymer network, 4 wt %. of the photoinitiator (Irgacure 1173) was added to the Ebecryl 150 resin. Afterwards, various amount of fillers (TP or TGP) were added to produce the expected percentage ratio of the filler in the UV-curable resin. The blends were mixed with a glass baguette to obtain homogenous dispersions. After the procedure, the mixtures were coated on glass slides and spread using a wire wound applicator (thickness set to 200 µm). To start the process of curing, slides were exposed to the UV radiation (300W; FusionUV Systems, Inc., Gaithersburg, MD, USA) in Qurtech machine model QSB-6 (Aspelt, Luxembourg) working in the horizontal configuration. The lamp-sample distance was fixed at 10 cm. Applied time of 15 s was sufficient to complete the photo-polymerization reaction. The obtained UV-cured membranes were carefully peeled off from the glass substrate and used for further analysis. 

### 2.2. Membrane Characterizations

Optical micrographies were taken with optical microscope Nikon Eclipse LV (European Headquarters Nikon Instruments Europe BV, Amsterdam, Netherlands). The membrane’s morphology was characterized by environmental scanning electronic microscopy (ESEM), using a FEI Quanta 600 (Thermo Fisher Scientific, Waltham, MA, USA). The samples were cryofractured into liquid nitrogen and then fixed on the support suitable for the cross-section analysis. Membrane thickness was measured by ESEM on the sample cross-section at least in three points.

The organic powders and the blended-resin systems were examined by infra-red attenuated total reflectance spectroscopy (IR-ATR) spectroscopy using a spectrometer (Nicolet 5700, Thermo Fisher Scientific, Waltham, MA, USA). Spectra were collected using 32 scans within the spectral range of 700–4000 cm^–1^ wavenumber at resolution of 1 cm^–1^. Thermal stability of the films was monitored by using a thermogravimetric analyser Discovery TGA (TA Instruments—Div. di Waters SpA, Sesto San Giovanni, Italy). All experiments were performed with samples of 10–12 mg in open alumina crucibles in the temperature range of 50–700 °C under a heating rate of 10 °C/min in nitrogen or air atmosphere. The values of T_10%_ (the temperature corresponding to 10% weight loss) and T_max_ (the temperature of maximum weight loss) were obtained by analysing the plots (TG) and computing the first derivative (dTG).

Differential scanning calorimetry (DSC) measurements were performed on TA Instruments Q20 DSC TGA (TA Instruments—Div. di Waters SpA, Sesto San Giovanni, Italy) using standard cell FC under nitrogen atmosphere with a 50 mL/min flow rate. Samples of 6–8 mg were sealed in aluminum crucibles. Three cycles were performed: first, samples were heated from 0 to 120 °C at a constant rate of 10 °C/min; secondly, samples were cooled down to 0 °C at 10 °C/min; finally, samples were again heated from 0 to 120 °C at a constant rate of 10 °C/min. 

The effect of the temperature on the storage modulus (log E’) and tan δ (loss tangent = E”/E’) was evaluated from dynamic-mechanical analyses (DMTA) were carried out on a DMA Q800 (TA Instruments – Div. di Waters SpA, Sesto San Giovanni, Italy) apparatus. The samples were cut to the rectangular dimension of 0.5 cm × 3 cm. The analysis was carried out in tensile configuration from room temperature to 120 °C with a heating rate of 5 °C/min; 1 Hz tension frequency and 0.05% of oscillation amplitude in strain-controlled mode were used. For each sample DMTA tests were repeated at least 3 times on different films.

### 2.3. Determination of Polyphenols Content

A total of 0.5 mL of the sample was mixed with 0.5 mL of Folin–Ciocalteu’s reagent and 10 mL of water. After 5 min, 8 mL of 7.5% (*w*/*v*) aqueous Na_2_CO_3_ solution was added to the mixture and stored for two hours in the dark. The concentration of released phenols was determined by UV-Vis spectroscopy at 765 nm using a UV-1800 Shimadzu apparatus (Thermo Fisher Scientific, Waltham, MA, USA) [28]. The results were calculated as Gallic Acid Equivalents (GAE), using a standard curve obtained from previous experiments: Abs = 0.1214x + 0.1142, where Abs is the absorbance of the sample at 765 nm and x is the phenolic concentration in µgGAE/mL (R^2^ = 0.9988). All samples were analysed in triplicate.

### 2.4. Determination of Flavonoids Content

A total of 0.5 mL of the sample was mixed with 0.5 mL of 2% (*w*/*v*) ethanolic solution of AlCl_3_. The mixture was stored for one hour in the dark. The concentration of released flavonoids was determined by UV-Vis spectroscopy at 420 nm using a UV-1800 Shimadzu apparatus (Thermo Fisher Scientific, Waltham, MA, USA). The results were calculated as quercetin equivalents (QE), using a standard curve obtained from previous experiments: Abs = 0.0674x + 0.0182, where Abs is the sample absorbance at 420 nm and x is the phenolic concentration in µgQE/mL (R^2^ = 0.9955). All samples were analysed in triplicate.

### 2.5. Release Tests of Polyphenols and Flavonoids

A set of membranes cut to the dimension of 0.5 cm × 0.5 cm were placed in glass vials containing 4 mL of water. The physical parameters of the release depend on several factors, i.e., not only on properties of the films such as thickness of the material, diffusion through pores, degradation of polymer matrix, but also for mechanical cracks or physical hindrance. To exclude the last ones, each membrane was cut with particular attention and hang in the vial to have the maximum contact with the liquid. The films were supported with a plastic ring that allowed them to get into maximum contact with the liquid. For each filler, a set of four vials with different equilibrium time were analysed: 0.5, 1, 6 and 24 h. After the desired time, each membrane was discarded and the solution analysed spectrophotometrically according to the procedures explained below. As a reference of total release, a vial with the propolis amount corresponding to the one contained in 5%, 10% and 15% samples, respectively, was exposed to the mixture EtOH-H_2_O (7:3 *v*/*v*) for 24h. Filtered solutions were used for further analysis.

### 2.6. Antibacterial Activity

The antibacterial activity for the best performing membranes was studied against *Staphylococcus epidermidis* (*S. epidermidis*, strain CECT231). The experiments consisted in the cultivation of bacteria at 37 °C during 24 h in 5 mL Nutrient broth (NB, CECT1) medium. After that time, the cultures were 1/5 diluted in NB medium. The NB was prepared by dissolving 5.0 g/L of beef extract, 10.0 g/L of peptone and 5.0 g/L of sodium chloride in distilled water. One square centimetre membranes ET20, EP10 and EP15 were immersed in NB medium. *S. epidermidis* was inoculated and incubated for 1, 2, 3, 4, 6, 8 and 24 h at 37 °C. Positive and negative controls were also prepared. After each time 25 µL sample of bacteria solution was placed on the surface of NB-agar solid medium plate and incubated at 37 °C for 24 h. NB-agar solid medium was prepared by dissolving 3.75 g of beef extract, 7.5 g of peptone, 3.75 g of sodium chloride and 11.25 g of agar in distilled water. Solid medium was poured onto a plastic Petri dishes and left for gelation. Experiments were performed in duplicate.

## 3. Results and Discussion

A set of eight membranes were prepared as stated in Table 1. Two different sources of biologically active compounds were used: *Galla chinensis* tea powder (TP) and Taiwanese green propolis (TGP). 

**Figure 2:**
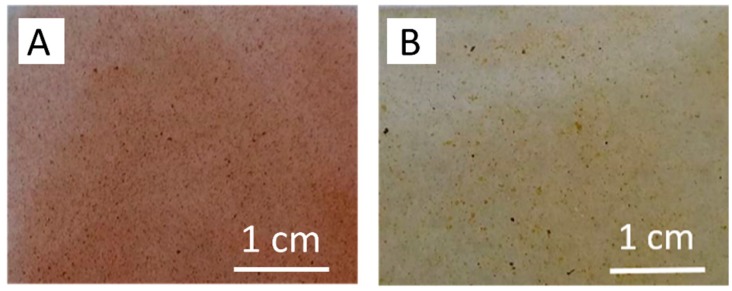
Optical micrographics of ET10 (**A**) and EP10 films (**B**).

Membranes ET5–ET20 were prepared with 5, 10, 15 or 20 wt % of TP, while membranes EP5, EP10, EP15 were obtained from Ebecryl 150 resin loaded with 5, 10 and 15 wt % of TGP, respectively. Membrane E0 was fabricated without a filler. Unfortunately, due to characteristic of propolis [29], which contain high amount of wax, films containing propolis weight percentage higher than 15 exhibited very poor mechanical properties and were not suitable for further analysis. Optical microscope studies put into evidence that in all cases both fillers are homogenously distributed in the membrane structures. As a matter of example, Figure 2 presents photos of ET10 and EP10 film surfaces captured by an optical microscope. 

**Figure 3:**
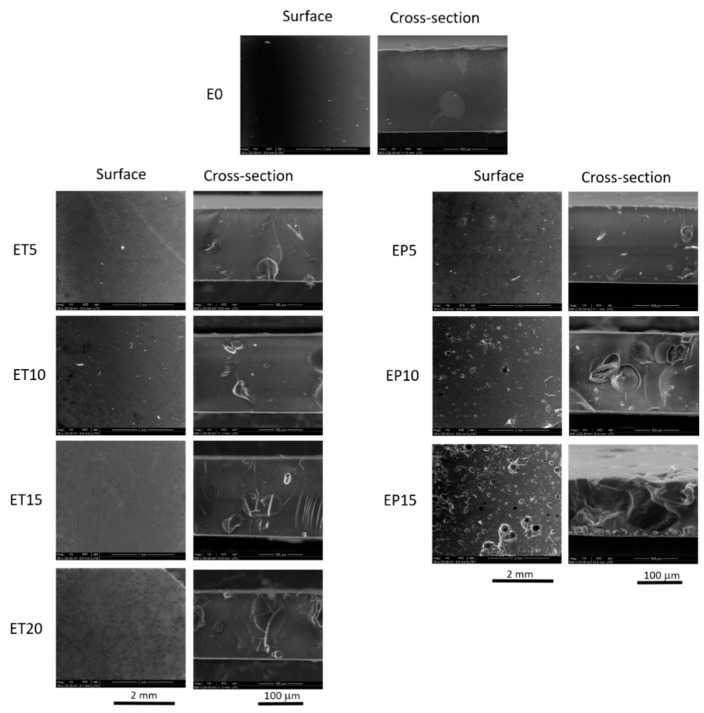
ESEM micrographs of membrane surfaces and cross-sections.

Figure 3 shows membrane surfaces and cross-sections captured by means of ESEM microscope. E0 membrane possesses dense structure, while in case of the composite films, the presence of the fillers can be easily evidenced in their cross-sections. Furthermore, the ESEM cross-section micrographs show that both TP and TGP particles are well incorporated in the membrane structures and they form a part of compact films. Surfaces of the membranes prepared with tea powder seem to be smooth while in case of EP membranes the TGP particles can be observed on the membrane surfaces. It is noteworthy that the particle size of the TP filler is in a range of 5–30 µm and it is twice smaller than TGP size. Moreover, the particle sizes of the fillers influence the membrane thicknesses. Indeed, thickness of the EP membranes is approximately 6% larger than the EP membranes. In the case of EP15, the ESEM cross-section image also suggests the existence of larger TGP aggregates and a less homogenous distribution in EP15.

**Figure 4:**
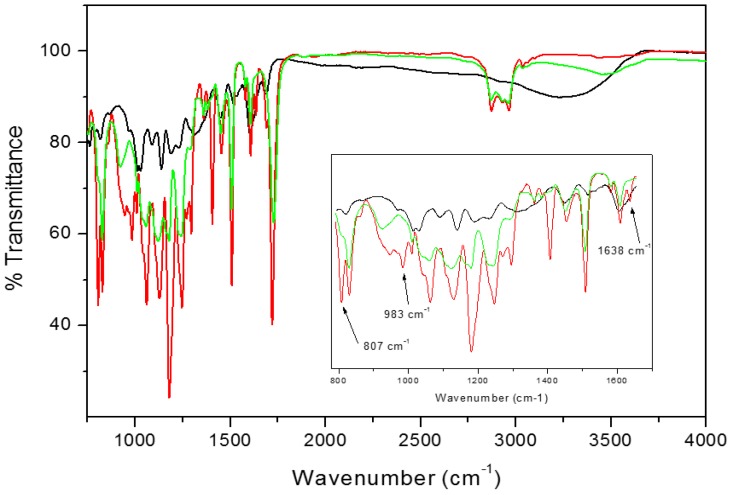
FTIR spectra of tea powder (black line), ET10 before UV-curing (red line) and ET10 UV-cured (green line). In the inset, the 790–1650 cm^−1^ region is zoomed.

**Figure 5:**
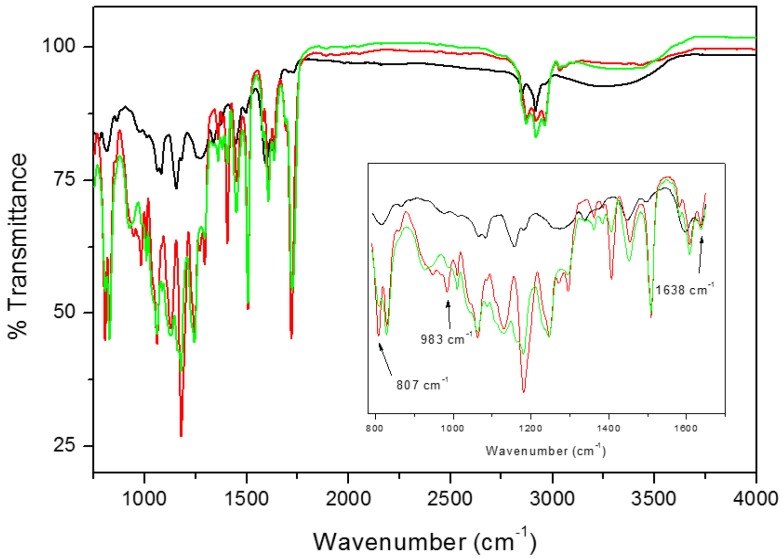
FTIR spectra of Taiwanese green propolis (TGP) powder (black line), EP10 before UV-curing (red line) and EP10 UV cured (green line). In the inset, the 790–1650 cm^–1^ region is zoomed.

IR analysis was performed to check the occurrence of UV curing and the filler presence inside the membranes. As a matter of example, Figure 4 shows the IR spectra of tea powder, ET10 sample uncured and after UV curing, respectively, while Figure 5 shows the IR spectra of propolis powder, EP10 sample uncured and after UV curing, respectively. From the comparison of the spectra of the resins before (red lines) and after UV-curing process (green lines), it is noticeable that the peak at 1638 cm^–1^, ascribed to the acrylic C=C stretching and the peaks at 983 cm^–1^ and at 807 cm^–1^, characteristic of =C–H stretching in acrylic resins, completely disappear on curing or it is strongly reduced (see inset in Figure 4 and Figure 5). For sake of comparison, Figure S1 reports the FTIR spectra of neat Ebecryl before and after UV curing in the same conditions, respectively. This observation confirms that the UV-curing process was driven to completion [30] in the case of ET systems; nevertheless, some residual reactivity can be still supposed in the case of the membranes containing TGP. No evident changes in other peaks of the original resin could be observed, which suggests that only physical interactions are established between Ebecryl and the fillers; this is desirable if their efficient release from the membrane is sought. 

It is well known that both tea and propolis are rich in polyphenols and flavonoids, which are clearly visible in their FTIR spectra [16,31]. In the black lines of Figure 4 (TP) and Figure 5 (TGP), the wide peak situated between 3000 and 3600 cm^–1^ is related to the –OH group in molecules forming inter- and intramolecular hydrogen bonds. Additionally, propolis powder (Figure 4, black line) contains discrete bands situated around 2880–2930 cm^–1^, attributed to –OH groups which stay in accordance with previously reported data [16,24]. The fingerprint region of both compounds is similar and possess IR bands at about ~1600 cm^–1^, 1450 cm^–1^ and 1100 cm^–1^, referring to C=C and C=O groups due to aromatic rings stretching vibrations of polyols, flavonoids and amino acids [16,24,32]. Both systems after UV-curing (green lines) possess bands belonging to polyphenols (3000 and 3600 cm^−1^) and acrylic resin (700–1800 cm^–1^). From the results, we can conclude that both fillers were successfully incorporated into the cured resin.

Thermogravimetric (TGA) analysis was used to get information about the amount and rate of decomposition of the samples as a function of temperature in a controlled atmosphere (nitrogen or air). T_10%_, Tmax and the percentage of the residue at 700 °C of all the samples in both atmospheres are gathered in Table 2, while the corresponding TG and dTG graphs are depicted in Figures S3 and S4. 

As far as the fillers (tea and propolis) are concerned, the difference in the percentage of residues with or without access to oxygen is clearly visible (Table 2 and Figures S1 and S2). In both cases, an air atmosphere accelerates the combustion over two times. In the set of synthesized films, the higher percentage of the residue with respect to the neat resin E0, both in aerobic and anaerobic conditions, is connected with the increased filler content. 

In nitrogen atmosphere, it seems that the presence of tea does not noticeably influence the degradation process of the resin (Figure S1). On analysing dTG graphs and comparing their Tmax values (Table 2), it can be seen that the addition of 20% tea (ET20 sample) lowers the maximum degradation temperature of Ebecryl (E0 sample) only of 4 °C, whereas the presence of 15% TGP (EP15) causes a decrease of 12 °C. On the other hand, T_10%_ is remarkably influenced, as expected on the basis of lower decomposition temperatures of the fillers.

More complex changes are observed in thermo-oxidative process (Figure S2). First peak observed in dTG curves situated between approximately 200 and 300 °C is most probably connected with the removal of moisture. The main decomposition steps occur between 300 and 600 °C. Actually, degradation of the films in aerobic atmosphere takes place in three steps [33]. The first step usually refers to the decomposition of small molecular weight compounds (i.e., dimers or trimers); the second step, the main one, is connected with the major degradation of the polymer network due to cross-links breaking. The last degradation step can be characterized as the oxidation of the degradation products created in the previously reported steps [33]. The presence of the organic fillers increases the onset temperature of the first degradation step as it can be easily noticed by comparing T_10%_ and T_max1_ values. Therefore, addition of both fillers slightly thermally stabilizes the polymer networks. However, on analysing the full set of membranes, higher fillers content leads to lowering of the temperature values corresponding to the following degradation steps.

Overall, incorporation of the organic fillers keeps the desirable characteristics of the UV-cured resin as far as thermal stability is concerned. However, the results obtained for EP15 film put into evidence the lack of T_max1_, whereas in case of tea this situation is observed for ET20 sample (Table 2), thus showing a change in the decomposition mechanism. This may suggest the maximum amount of loading which does not affect the polymer network in relevant extent. Lei et al. as well noticed a strong addition-dependent pattern [18]. In particular, the incorporation of tea polyphenols to pectin and konjac glucomannan films increased their mechanical and water-resistant properties but only to some extent. Higher concentrations (beyond 2%) had a negative impact on their performance.

Polymer glass transition temperature (T_g_) is a diagnostic value for determining compatibility or incompatibility of polymer blends. Table 3 presents the values of glass transition temperature (T_g_) determined by DSC for E0, ET and EP samples, while Figures S3 and S4 show their enthalpy relaxation curves. All samples were heated twice, as reported in the experimental section.

On analysing the first heating run, it is visible that addition of both fillers does not influence T_g_ of neat Ebecryl (E0 sample). Glass transition temperature of pure resin was determined as 49 °C and stays in agreement with previously reported data (50 °C) [33]. However, in the presence of the fillers, qualitative differences in plots are unquestionable (Figures S3 and S4). On the other hand, stronger changes are visible in the second heating run. In this case, all T_g_’s for polymers with various percentages of fillers are characterised by lower temperatures than neat resin sample E0 (Table 3). Actually, in the second heating scan, glass transition of E0 slightly increases (53 °C) whereas T_g_ of all blends decreases; furthermore, no pronounced thermal transition is observed. This suggests that after the first heating scan, the segments between cross-link points in the matrix were re-arranged. However, in this case the reduced T_g_ value of all blends in comparison with pure resin indicates higher matrix mobility: this could be related with higher amount of filler, that can act as a plasticizer for Ebecryl films. In any case, one should underline that no blend compositions evidenced multiple glass transition temperatures from DSC analysis over the entire range of scanning, thus suggesting that the tested samples were homogeneous. 

**Figure 6:**
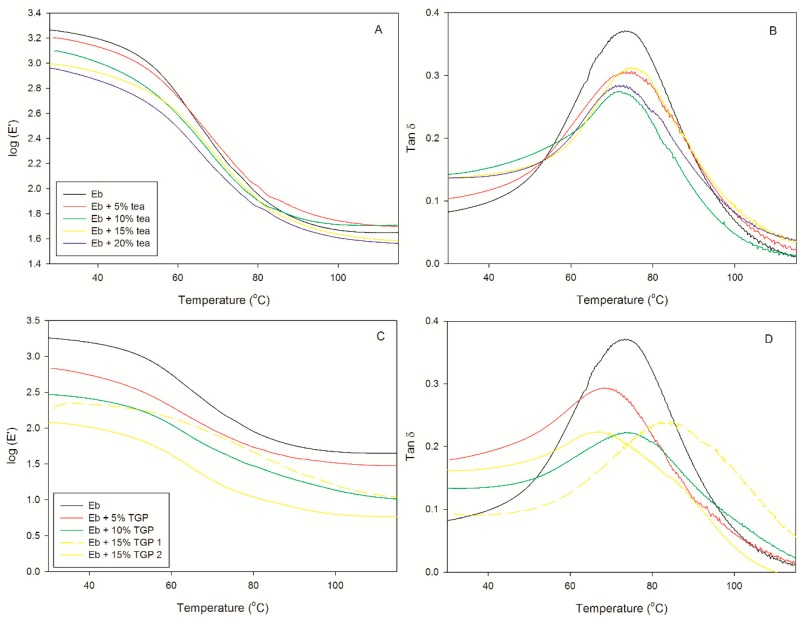
(**A**) Storage modulus and (**B**) tan δ (loss tangent = E”/E’) of tea films and (**C**) the storage modulus and (**D**) tan δ (loss tangent = E”/E’) of TGP films.

DMTA technique is often used to investigate the compatibilities between filler and polymer matrix [34,35]. In fact, by this technique it is possible to identify particle/polymer interactions and evaluate the reinforcement or labefaction effects on the viscoelastic properties of matrix. The dynamic-mechanical thermal properties of Ebecryl blends are shown in Figure 6 and the values of the determined T_g_ and storage modulus (E’) at 32 °C are reported in Table 3. As expected, the addition of both fillers (tea and TGP) affects the UV-cured acrylic resin: in each formulation, the storage modulus decreases and tan δ peak broadens and is slightly shifted. In both cases, the stiffness and the T_g_ of the blends decrease as a consequence of increasing percentage of the filler.

In the case of propolis, the influence on the viscoelastic properties of Ebecryl matrix is characterized by a gradual decrease in storage modulus over the whole temperature range and by a significant broadening and decrease in tan δ, as expected based on the plasticizing effect of TGP. Moreover, FTIR results reported in Figure 5 for EP10 suggest that the presence of increasing amounts of TGP interferes somewhat in the completion of crosslinking reaction. When the blend reaches 15 wt %, DMTA test does not provide clear results. Repeated analysis on different samples gave lower storage modulus and tan δ peak located at lower temperature as depicted in Figure 6C,D (yellow solid and dashed lines). In order to understand this evidence, we should take into account that, unarguably, higher content of TGP softens the polymer network. This situation is understandable when keeping in mind that natural propolis contains not only flavonoids and polyphenols, but also waxes. Indeed, several melting points were reported [29]. The two values reported for the maximum of tan δ peaks from tests on different samples (Figure 6D and Table 3) interestingly stay in agreement with the two transitions evidenced by DSC for TGP (Figure S4, blue line). On analysing the morphology of the membrane by ESEM (Figure 3), it can be seen that this formulation is characterized by less homogenous distribution and larger aggregates of filler in comparison to the blend obtained with tea. Therefore, it is reasonable to attribute the mismatch between different DMTA tests on EP15, to the heterogeneities present in this sample.

Summing up, DMTA results are in good agreement with DSC analysis debated earlier. Conducted experiments confirm that the mechanical properties of the prepared films strongly depend on their composition, as expected. In particular, the highest percentage of both fillers remarkably softens the polymer network. 

Table 4 gathers the total concentrations of polyphenols and flavonoids obtained for tea and TGP powder in the determinant test and treated as 100% of release. 

**Figure 7:**
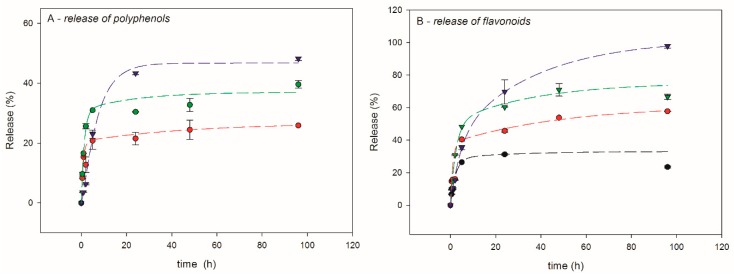
Tea release kinetics in water: (**A**) release of polyphenols and (B) release of flavonoids. Experimental results: black dots (ET5), red dots (ET10), green dots (ET15) and blue dots (ET20). The corresponding exponential fitting are shown by dashed lines.

The release kinetics of polyphenols and flavonoids for ET samples is shown in Figure 7.

Tea extract is soluble in water and the tea powder incorporated in the membrane passes fast into environment, as expected. As it can be observed in Figure 7, all presented graphs adopt the exponential shape and seem to reach a saturation. The fastest “burst” release is observed in the first 5 h and can be attributed to the filler trapped closer to the membrane surface; as shown by Figure 5, 24 h seem to be sufficient to reach a plateau for polyphenols measurement. In the case of ET20, release is the fastest one. In the case of ET5, experimental determination of released polyphenols gave low values affected by too high an error, and were therefore considered not significant. On the other hand, the membranes ET10, ET15 and ET20 after 96 h of experiment reached 26%, 39% and 48% of release, respectively. These results most likely arise because of the membrane loosening consequent to higher amount of filler, which eases solvent penetration into the membrane and the subsequent filler extraction. Indeed, the release kinetics of flavonoids shows that, after 96 h, as much as 98% of components is released from ET20 sample. Visibly lower released concentration was found from membranes containing lower filler amount: 67%, 57% and 23%, respectively, for ET15, ET10 and ET5. Very recently Gao et al. [36] presented a study of time release tests for polysaccharide films based on pectin and chitosan. They incorporated tea polyphenols and created formulations of 2.5%, 5% and 15% *w/w*. The highest ratio of 45% release was registered for 15% loading after 24 h.

**Figure 8:**
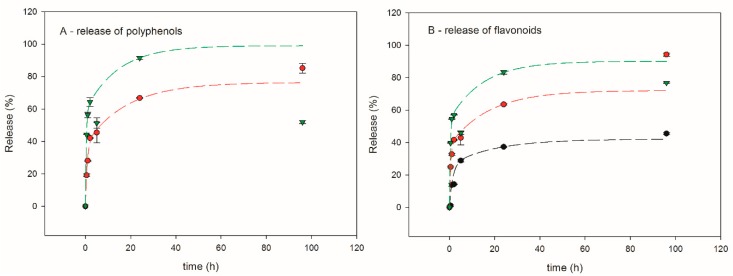
Propolis release kinetics in the mixture EtOH-H_2_O (7:3 *v*/*v*). (**A**) release of polyphenols and (**B**) release of flavonoids. Experimental results: black dots (EP5); red dots (EP10); green dots (EP15). The corresponding exponential fitting are shown by dashed lines.

Figure 8 reports the release kinetics of polyphenols and flavonoids in the mixture EtOH–H_2_O (7:3 *v*/*v*) for EP samples.

All EP films show similar kinetic release pattern of antioxidants through the whole time of the experiment (96 h), which is also analogous to the one observed for ET samples. Actually, also for EP samples the fastest release is observed within the first five hours. A similar time-dependent mode was observed for propolis extract release in natural rubber latex membrane [16]. In the same way as ET5, in the case of EP5 the experimental values of released polyphenols were affected by great uncertainty and were, therefore, discarded. On the other hand, the determination of flavonoids released form EP5 gave coherent values. During the first hour, EP10 membrane releases 28% of polyphenols and 32% of flavonoids; after 24 h, this amount rose to 67% and 64%, respectively. Increased amount of the filler to 15% increases the release of the active substance, i.e., after 24 h, 91% of polyphenols and 83% of flavonoids are being exposed to the environment. Similarly to ET systems, the results suggest that increased percentage of filler facilitates solvent penetration and enhances release. However, while in EP5 and EP10 after 96 h of experiment a plateau can be envisaged, this is not the case for EP15, since the release from this sample significantly decreased at this time, especially for polyphenols. It stays unclear why the experiment ended with lowered release value than expected. It could be explained with either spontaneous sedimentation or unexpected stacking effects of aromatic moieties of antioxidants. Nevertheless, obtained results are satisfactory from time-release point of view. Moreover, this unclear result somehow overlaps with the adverse effect of 15 wt % formulation onto mechanical and thermal properties of Ebecryl matrix.

Presented release results stay in agreement with already published data. Hydroalcoholic extract of propolis was incorporated into the poly(vinyl alcohol) (PVA) to create nanofiber mat (60% *w*/*w*) [23]. The initial burst within the first 2–3 h released around 45% of the substance. Later, a release of 69% and 83% for 24 h and 96 h, respectively, was observed.

Antibacterial activity was preliminarly tested against *S. epidermidis*. This is a staphylococcus bacterium, consisting of Gram-positive coconuts arranged in groups; it is a component of the normal microbiota of human skin and surface mucous membranes [37,38]. Nevertheless, it has also turned as the most important pathogen in infections related to some biomedical materials, such as joint prostheses and heart valves [39,40]. *S. epidermidis* provokes the growth of biofilms in plastic devices that are inserted in the body. Bacteria possess an outer layer of polysaccharides that adheres strongly to plastic, which also hinders antibiotic treatment, by preventing their penetration. For instance, together with other *Staphylococcus* bacteria, is responsible for 47% of joint prosthetic infections and probably represents the most common species found in laboratory tests [39].

**Figure 9:**
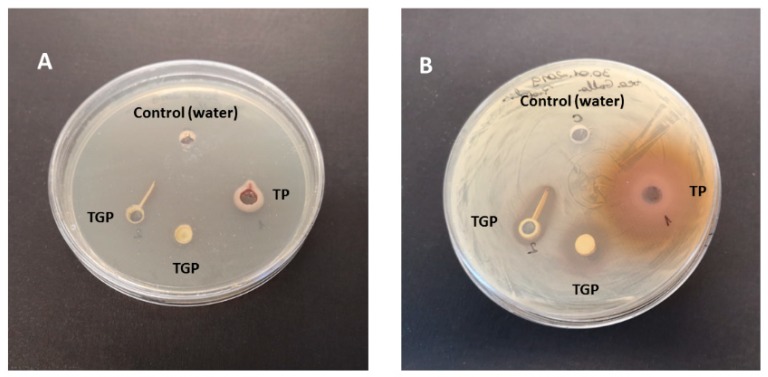
Inhibitory activity of TP and TGP extracts against *S. epidermidis.* (**A**) time 0; (**B**) after 24 h incubation.

First, we tested antibacterial activity of antioxidants (polyphenos and flavonoids), extracted from TP and TGP, respectively. Figure 9 shows the results obtained at time 0 (Figure 9A) and 48 h (Figure 9B), respectively. As it can be seen, both TP and TGP extracts show significant inhibitory activity against *S. epidermidis,* as evidenced by the clear holes surrounding the well after 48 h. It can be also noticed that TP exhibits remarkably higher diffusion into the medium.

**Figure 10:**
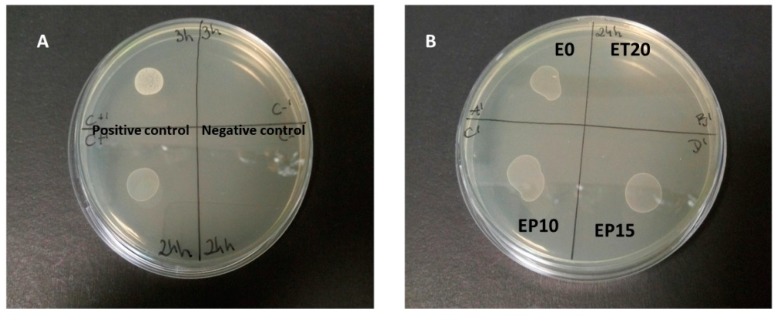
Antibacterial activity against *S. epidermidis* after 24 h incubation. (**A**) Positive control (liquid medium + *S. epidermidis)* and negative control (liquid medium without bacteria); (**B**) E0, ET20, EP10 and EP15 membranes.

Afterwards, we tested whether the antibacterial activity is sustained when the antibacterial agents are entrapped into a polymeric matrix, i.e., in the previously prepared membranes. For this scope, we selected the membranes with higher antioxidant content that is ET20, EP10 and EP15. Controls were also prepared: the positive one, containing the liquid medium + *S. epidermidis*, while the negative one contained the liquid medium without bacteria. In addition, the test was also performed with neat E0 membrane. The results are shown in Figure 10.

It can be noticed that, after 24 h incubation, E0 membrane shows no antibacterial effect, as expected, which also suggests that no component of UV formulation possesses any antibacterial activity after UV-curing. Actually, in the literature several examples about UV-cured films containing additives with antibacterial activity were reported; nevertheless, when the films containing no additive were investigated, no inhibitory effect could be found [41,42,43].

Differently, a clear inhibitory effect can be evidenced in the case of ET20 membrane. On the other hand, no inhibitory effect can be detected for EP10 and EP15 systems. In fact, this apparently surprising result can be reasonably explained: first, it must be pointed out that fractionation of propolis to get compounds is difficult due to its complex composition [44]. Traditionally, the soluble fraction in alcohol, called ’propolis balsam’ is extracted and used for therapeutic scopes. As a matter of fact, we could get TGP relatively fast released, when 7/3 *v*/*v* ethanol/water mixture was used as medium. Even in this case, from Table 4 one can observe that the obtained content of polyphenols and flavonoids from EP10 and EP15, is far lower than the one released from ET membranes containing the same amount of additive and extracted from water, thus putting into evidence lower solubility of TGP with respect to TP in their respective extraction media. Actually, it was reported that water extract from TGP revealed no antibacterial activity against *S. aureus* [7]. Therefore, since the antibacterial activity tests are performed in aqueous medium, the lack of inhibition of EP systems could be due to an insufficient amount of antioxidants diffused into it. 

## 4. Conclusions

We prepared acrylic membranes, based on Ebecryl 150 acrylic resin, filled with tea extract from *Galla chinensis* (TP) or Taiwanese green propolis (TGP) as organic fillers with potential antibacterial activity. The organic additive was added in different percentages, ranging from 5% to 20%. In all cases the presence of TP and TGP did not remarkably interfere with the UV-curing process and homogeneous, self-sustainable membranes could be obtained, as evidenced by FTIR and ESEM analyses; however, in the case of 20 wt % TGP, the resulting membrane showed poor mechanical properties and was discarded. The desirable characteristics of the UV-cured acrylic resin were sustained in most cases, as shown by TGA, DSC and DMTA. However, the concentration dependent motif is clearly visible. Results obtained for 15% TGP film and 20% TP suggest that this may be the maximum amount of the filler, which does not affect the polymer network and its mechanical properties in higher extent. Detailed release tests of polyphenols and flavonoids revealed that all sets of membranes show similar kinetic release pattern of antioxidants through the experiment. The fastest release is observed within first 5 h and for all systems 24 h seem to be sufficient to achieve a plateau. For all the investigated membranes, the release rate increase with increasing the tea or propolis content in the matrix. Finally, preliminary antibacterial tests were performed against *Staphylococcus epidermidis* on the membranes containing higher additive content and revealed a satisfactory inhibitory effect in the case of 20% TP membrane. On the other hand, no effect was detected for TGP containing membranes, probably due to the limited solubility of the additive into the aqueous medium. 

## Figures and Tables

**Table 1:** Filler content of the obtained acrylic membranes.

Membrane	Filler	wt %	Thickness (µm)
E0	-	-	163.6 ± 0.3
ET5	Tea powder (TP)	5	168.2 ± 1.9
ET10	Tea powder (TP)	10	174.2 ± 1.8
ET15	Tea powder (TP)	15	181.2 ± 2.1
ET20	Tea powder (TP)	20	193.1 ± 2.4
EP5	Taiwanese green propolis (TGP)	5	178.2 ± 0.3
EP10	Taiwanese green propolis (TGP)	10	184.5 ± 1.0
EP15	Taiwanese green propolis (TGP)	15	196.5 ± 0.9

**Table 2:** Gathered values of T_10%_, T_max_ and the percentage of the residue at 700 °C obtained from thermogravimetric analysis for neat Ebecryl resin, ET and EP films.

Nitrogen (N_2_)				Air				
Sample	T_10%_ (°C)	T_max_ (°C)	Residue at 700 °C (wt %)	T_10%_ (°C)	T_max1_ (°C)	T_max2_ (°C)	T_max3_ (°C)	Residue at 700 °C (%)
E0	397	440	2.95	343	382	445	544	0
TP	209	226, 264	40.82	214	222	467	499	1.09
ET5	386	437	5.14	349	386	433	537	0
ET10	323	436	6.62	325	388	432	536	0.08
ET15	305	436	8.81	320	387	430	520	0.13
ET20	269	436	10.00	271	-	427	526	0.14
TGP	258	309, 334	31.30	256	282	379	512	1.33
EP5	380	435	5.06	357	388	433	539	0.04
EP10	333	429	6.23	332	383	431	533	0.16
EP15	323	428	7.79	316	-	430	530	0.29

**Table 3:** T_g_ and storage modulus (E’) obtained from DSC and DMTA experiments. N.d.: not determined.

Sample	DSC		DMTA	
	T_g_ (°C) run 1	T_g_ (°C) run 2	E’ T = 32 °C (MPa)	T_g_ (°C)
E0	49	53	1789 ± 15	74 ± 0.5
TP	69	N.d.	-	-
ET5	47	48	1461 ± 114	75 ± 3
ET10	49	46	1110 ± 140	73 ± 1
ET15	49	47	940 ± 16	76 ± 1.5
ET20	49	43	800 ± 73	74 ± 3
TGP	58, 82	47, 59	-	-
EP5	49	48	696 ± 25	71 ± 1.5
EP10	49	48	232 ± 49	77 ± 2
EP15	52	51	149 ± 49; 56 ± 2	87 ± 3; 71 ± 3

**Table 4:** Total concentration of polyphenols and flavonoids released from powder samples of tea or TGP in water or in EtOH-H_2_O (7:3 *v/v*), respectively, after 24 h. The sample name refers to the membrane containing the same total amount of tea or TGP powder.

Corresponding Membrane	Polyphenols (µg GAE/mL)	Flavonoids (µg QE/mL)
ET5	105.11 ± 0.44	19.27 ± 0.02
ET10	201.9 ± 13.6	26.7 ± 0.5
ET15	311.1 ± 1.3	35.4 ± 2.9
ET20	472.48 ± 0.36	30.52 ± 0.24
EP5	71.0 ± 3.1	12.37 ± 0.04
EP10	138.9 ± 0.9	18.32 ± 0.02
EP15	194.2 ± 1.1	25.72 ± 0.04

## Supplementary Materials

The following are available online at https://www.mdpi.com/2073-4360/12/2/358/s1, Figure S1. FTIR spectra of neat Ebecryl 150. Figure S2. Thermogravimetric analysis performed in nitrogen atmosphere. Figure S3. Thermogravimetric analysis performed in air atmosphere. Figure S4. DSC curves (1^st^ and 2^nd^ heating runs) for E0, tea and ET samples at a heating rate of 10 °C/min. Figure S5. DSC curves (1^st^ and 2^nd^ run) for E0, TGP and EP samples at a heating rate of 10 °C/min.
