# Supplementary material for: Preparation and Characterization of UV-Curable Acrylic Membranes Embedding Natural Antioxidants
Source: Polymers (Basel). 2020 Feb 6;12(2):358. doi: 10.3390/polym12020358 (PMC7077452; doi:10.3390/polym12020358)
Supplement: Supplementary file 1 [file polymers-12-00358-s001.pdf]

Supplementary Material

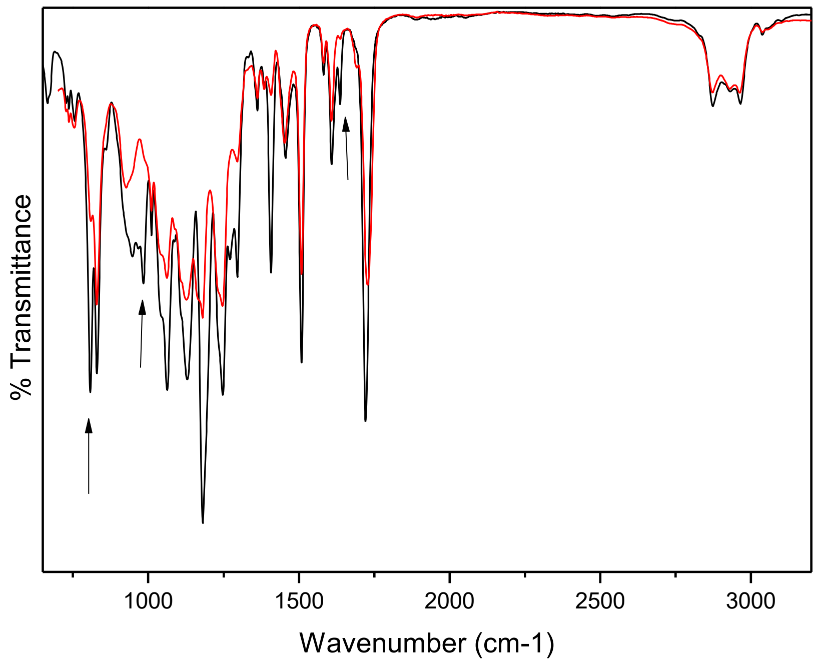

Figure S1 FTIR spectra of neat Ebecryl 150: black line, uncured; red line, after UV curing. The arrows highlight the characteristic bands of acrylic groups.

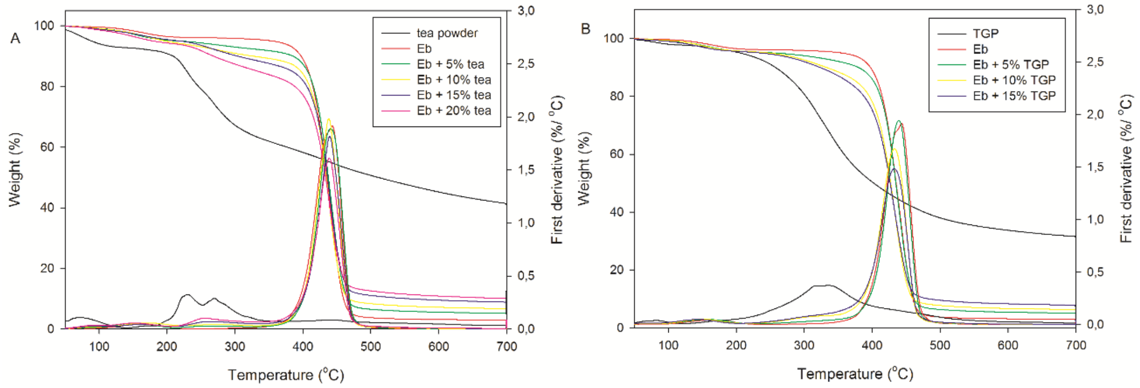

Figure S2 Thermogravimetric analysis performed in nitrogen atmosphere. TG and dTG curves of E0, ET (A) and EP (B) films.

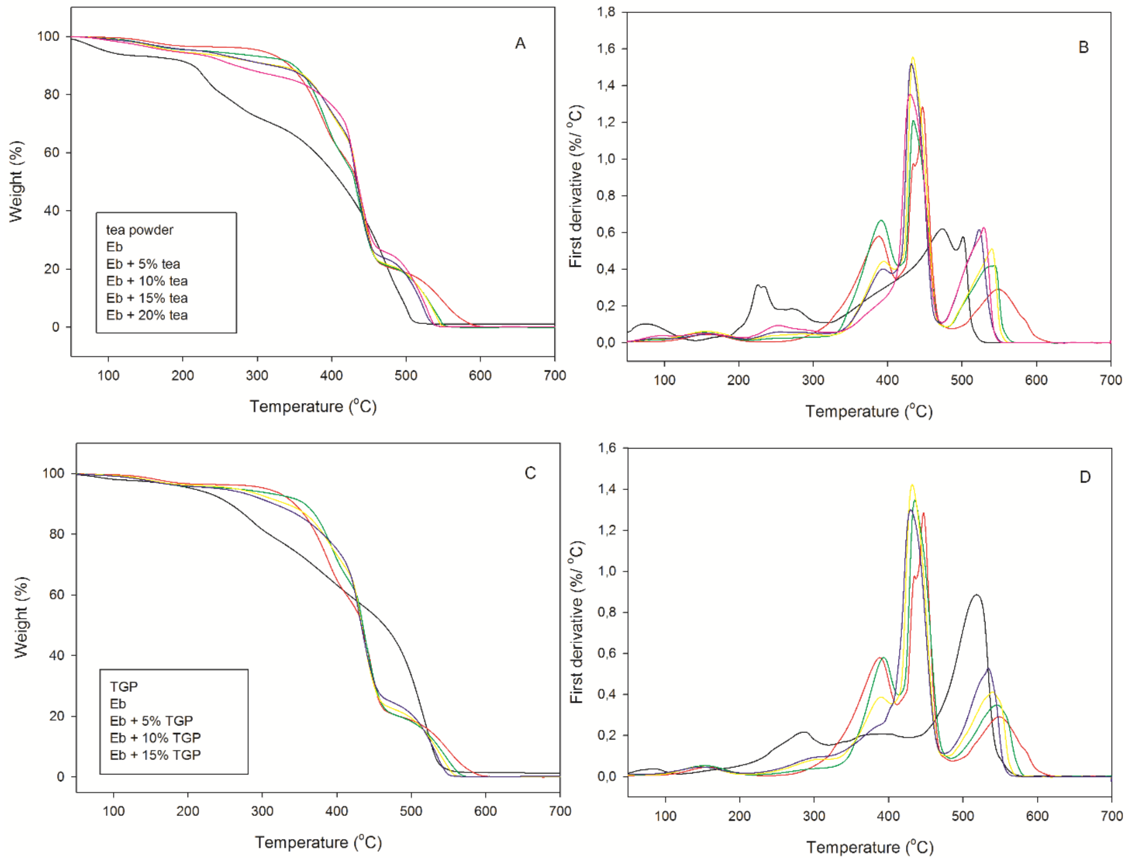

Figure S3 Thermogravimetric analysis performed in air atmosphere. (A) TG and (B) dTG curves of E0 and ET films. (C) TG and (D) dTG curves of E0 and EP films

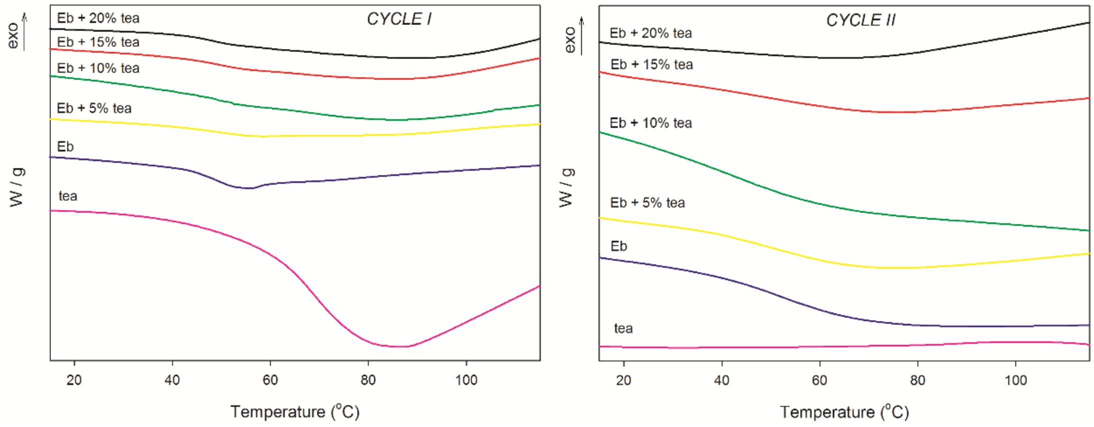

Figure S4. DSC curves (1st and 2nd heating runs) for E0, tea and ET samples at a heating rate of 10°C/min.

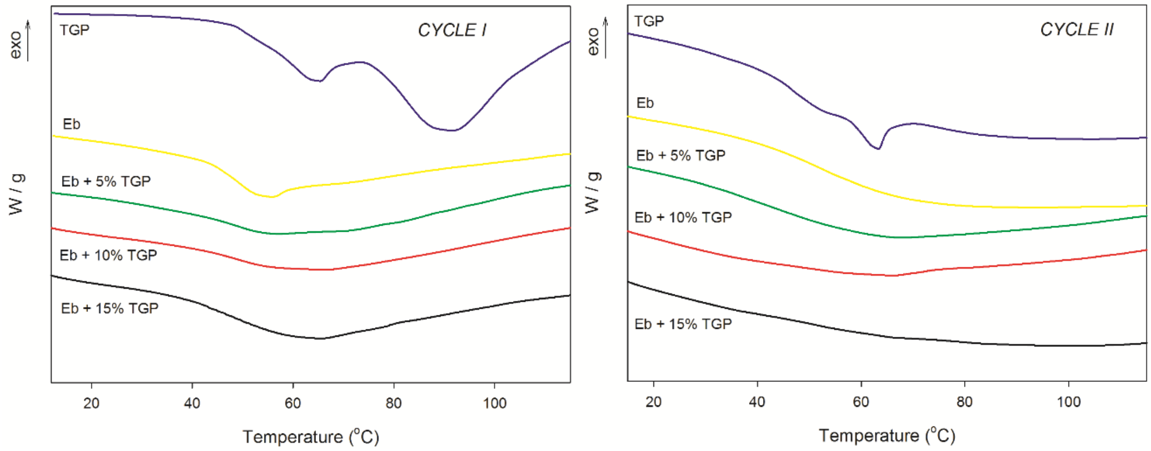

Figure S5. DSC curves (1st and 2nd run) for E0, TGP and EP samples at a heating rate of 10°C/min.
